# Microbial inoculation enhanced the thermophilic phase and improved final maturity-related properties of coffee husk composting for tomato seedling substrate production

**DOI:** 10.3389/fpls.2026.1906101

**Published:** 2026-09-09

**Authors:** Fang Liu, Ruixiang Zhu, Qi He, Shiwen Qin, Feifei He

**Affiliations:** 1 School of Agriculture, Yunnan University, Kunming, China; 2 Yunnan Provincial Key Laboratory of Coffee, Baoshan, Yunnan, China

**Keywords:** coffee husks, composting, growth medium, microbial agents, microbial diversity, physicochemical properties

## Abstract

Coffee husk composting is constrained by high lignocellulose content, slow decomposition, and residual phytotoxicity. Whether microbial inoculation can accelerate this process while producing a horticultural growing medium remains unclear. We compared coffee husks composted with a commercial microbial agent (a consortium of photosynthetic bacteria, lactic acid bacteria, yeast, filamentous fungi, and actinomycetes) (CHM) versus without (CH) over 180 days. The CHM treatment reached a higher peak temperature (55.6 °C vs. 43.5 °C) and at the end of composting achieved a numerically greater seed germination index (93.2% vs. 71.2%), indicating enhanced maturation and reduced phytotoxicity. High throughput sequencing showed that CHM did not drastically alter overall microbial α diversity but the final compost exhibited a marked reduction in the relative abundance of the genus *Fusarium* compared to CH. Beneficial bacterial genera such as *Pseudomonas*, *Sphingomonas*, and *Alcaligenes* showed a non-significant but consistent trend toward enrichment in CHM. When the mature CHM compost was blended with vermiculite, perlite, and decomposed cow manure, blends with lower compost inclusion (1:5 or 1:3 volume ratio of CHM compost to other components) supported tomato seedling growth comparable to a conventional peat based medium. These results demonstrate that microbial inoculation promote a more intense thermophilic phase and enhances final maturity in coffee husk composting, yielding a product with potential to partially substitute for peat in horticultural substrates. This approach offers a practical strategy for recycling coffee processing byproducts into a compost-based growing medium component.

## 1 Introduction

Global coffee consumption is projected to increase substantially by 2050, generating massive amounts of coffee husks as a processing byproduct (Millard, 2017). Most husks are landfilled or used only for low-value applications such as animal feed or combustion (de Oliveira Fernandes et al., 2025; Saxena et al., 2025). Yet coffee husks contain considerable organic matter and nutrients. Composting offers a sustainable route to convert this waste into a stable, soil-like amendment (Insam and de Bertoldi, 2007; Wang et al., 2023). However, coffee husk composting is inherently slow primarily because of its recalcitrant lignocellulosic matrix and phenolic compounds that inhibit microbial activity (Shemekite et al., 2014). Direct application of raw coffee husks to agricultural soils can suppress plant growth due to the leaching of these phytotoxic compounds (Shemekite et al., 2014). Previous efforts have focused on co-composting with nitrogen-rich additives such as poultry manure or urea to balance C/N, or on optimizing aeration and moisture (Dadi et al., 2019). These approaches improve decomposition rates but often neglect the microbial ecological mechanisms that drive process efficiency and end-product functionality.

Microbial inoculants have shown promise in accelerating lignocellulose degradation in other agricultural wastes, such as rice straw and olive pomace, by introducing cellulolytic or thermophilic taxa (Chukwuma et al., 2021; Jusoh et al., 2013; Wongfaed et al., 2023; Zhou et al., 2023; Zhu et al., 2018). A recent meta-analysis further confirmed that microbial inoculation significantly increases total nitrogen, total phosphorus, humification, and the germination index, while reducing cellulose, hemicellulose, and lignin content, compared to uninoculated controls (Nigussie et al., 2021). Moreover, advanced microbial agents consist of diverse functional strains including cellulose-degrading microorganisms, lignocellulose-degrading fungi and odor-suppressing microbes. Benefiting from synergistic interactions among these strains, the agents exhibit superior stability and environmental adaptability, which accelerates lignocellulose degradation via secretion of cellulases and lignin peroxidases, while lowering the emissions of greenhouse gases and malodorous gases (Zhou et al., 2025). Despite these advances, the role of microbial inoculation in coffee husk composting remains poorly understood, especially concerning long-term effects on microbial community dynamics, potential pathogen suppression, and the practical utility of the final compost as a plant growing medium. Most studies stop at assessing compost maturity and seldom test the formulated product in horticultural systems. This gap is critical because the ultimate value of any compost lies not only in its stability and safety but also in its ability to replace non-renewable inputs such as peat.

Peat remains the dominant component of growing media in soilless horticulture, but peat extraction destroys fragile peatland ecosystems and releases greenhouse gases (Picca et al., 2023). Approximately 90 million m³ of peat is extracted annually, with peatlands storing between 21% and 33% of global organic soil carbon stocks on less than 3% of the terrestrial surface (Picca et al., 2023). Finding renewable alternatives to peat is therefore a priority for sustainable horticulture. Coffee husk compost, if properly matured, could serve as a candidate, but evidence linking its microbial ecology to plant growth performance is lacking. Recent studies have shown that spent coffee grounds co-composted with biochar can partially replace peat in tomato substrates, though the results are strongly dilution- and plant development stage-dependent (Picca et al., 2023). Furthermore, the potential of microbial inoculation to suppress potential fungal pathogens during coffee husk composting has not been systematically investigated, despite the common occurrence of *Fusarium* species in organic wastes (Ekwomadu and Mwanza, 2023). Composts are now recognized not only as soil conditioners but also as reservoirs of antagonistic microorganisms that can inhibit fungal pathogens through the production of antifungal metabolites and competition for nutrients (Salman et al., 2024; Mironov et al., 2023). Inoculation with compost-indigenous antagonistic bacteria such as *Bacillus mojavensis* and *Pseudomonas aeruginosa* has been shown to significantly improve the antifungal activity of compost and enhance disease suppression (Zhao et al., 2023).

This study addressed two knowledge gaps: (1) the lack of comparative data on microbial inoculant efficacy in coffee husk composting versus traditional composting without an agent, and (2) the underexplored potential of coffee husk compost as a growth substrate for vegetable seedlings. In contrast to previous work that primarily optimized C/N ratios or co-composting materials, our study integrates microbial inoculation with comprehensive community profiling and direct horticultural performance testing. We specifically examine the underlying microbial mechanisms, including potential pathogen suppression and accelerated maturation, and translate these into a practical peat-substitute formulation.

We hypothesized that adding a microbial agent would improve functional microbial diversity, accelerate lignocellulose decomposition, enhance compost maturity, reduce phytotoxicity, and produce a material suitable for blending into a peat substitute. To test this, we established two treatments: coffee husks with a microbial agent (CHM) and coffee husks alone (CH). We monitored temperature, physicochemical properties, and microbial communities (16S and ITS amplicon sequencing) over 180 days. The mature CHM compost was then formulated with vermiculite, perlite, and cow manure at different ratios and tested for tomato seedling growth. By linking microbial changes to process performance and final product quality, this work aimed to provide a practical strategy for valorizing coffee husks in circular agriculture.

## 2 Materials and methods

### 2.1 Experimental materials and design

Coffee husks were collected from Baoshan, Yunnan Province, South China. Fresh husks were soaked in tap water (1:1, v/v) for 30 minutes, drained, and the procedure repeated until the soaking solution turned pale yellow, removing soluble phenolic compounds. The rinsed husks were dried and crushed to 0.5 cm particle size. Aerobic composting was conducted in three independent 60 L foam reactors (working volume ~50 L) per treatment, arranged in a completely randomized design within the greenhouse. A completely randomized design within the greenhouse. Ambient temperature ranged from 25 to 30 °C during the experiment. The initial C/N ratio was adjusted to approximately 25:1 by supplementing with urea. The total nitrogen concentration, pH, electrical conductivity (EC), and total organic carbon (TOC) of the raw husks were 15.0 g kg^−1^, 5.32, 2.14 dS m^−1^, and 384.4 g kg^−1^, respectively. Initial moisture was adjusted to 60–65% (w/w). The piles were turned manually every seven days to ensure adequate aeration.

Two treatments were established in triplicate: (1) coffee husks plus 0.3% (dry weight basis) microbial agent (CHM), and (2) coffee husks alone (CH). Each treatment had three replicate reactors (n = 3). The microbial agent (EM-composting inoculant, Shandong Junde Biotechnology Co., Ltd., China) is a commercial product containing a consortium of photosynthetic bacteria, lactic acid bacteria, yeast, fermentation filamentous bacteria, and actinomycetes. The microbial agent was incorporated into the raw materials at a rate of 0.3% (w/w). This consortium was selected based on its documented ability to produce hydrolytic enzymes, potentially suppress pathogens through competitive exclusion and antibiotic production, and accelerate organic matter stabilization. A schematic diagram of the experimental design is shown in **Supplementary Figure S1**.

### 2.2 Physicochemical analysis

Temperature was recorded daily at 10:00 AM using a digital thermometer inserted into the center of each pile at 30 cm depth. For physicochemical analyses, one composite sample was taken from each reactor at each sampling time (30, 60, 90, 120, 150, and 180 days) by thoroughly mixing subsamples collected from three different locations within the reactor. pH and EC were measured in a 1:10 (w/v) compost to deionized water extract using a pH meter (Model FE28-Standard, Mettler Toledo, Switzerland) and conductivity meter (Model DDS-307A, Leici, China), respectively (Uysal et al., 2019). Total organic carbon was determined by the potassium dichromate oxidation method (Shetty and Goyal, 2022). Total nitrogen was measured by the Kjeldahl method (Bremner, 1960). The C/N ratio was calculated as the mass ratio of total organic carbon to total nitrogen. For the seedling substrate experiment, all physicochemical parameters were measured using the same procedures.

### 2.3 High throughput sequencing and data processing

Samples for microbial analysis were collected at 30-day intervals: 30 days (D1, early stage), 60 to 120 days (D2–D4, middle stage), and 150 to 180 days (D5–D6, late stage). One composite sample from each of the three replicate reactors per treatment was used for DNA extraction at each time point (n=3). Samples were sieved (2 mm), and stored at −80 °C. Total genomic DNA was extracted using a PowerSoil DNA Isolation Kit (MoBio Laboratories, Carlsbad, CA, USA) according to the manufacturer’s instructions.

Bacterial 16S rDNA V5–V7 region was amplified with primers 799F (5′ AACMGGATTAGATACCCKG 3′) and 1193R (5′ ACGTCATCCCCACCTTCC 3′). Fungal ITS1 region was amplified with ITS1F (5′ CTTGCTCATTTAGAGGAAGTAA 3′) and ITS2 (5′ GCTGCGTTCTTCATCGATGC 3′). PCR products were purified, quantified, and sequenced on an Illumina NovaSeq platform (PE250).

Raw reads were quality-filtered, and amplicon sequence variants (ASVs) were inferred using the DADA2 pipeline. Taxonomy was assigned against Silva 138 (bacteria) and Unite 8.0 (fungi) databases. Niche width was calculated using the Levins measure (Levins, 1968) implemented in the R package “spaa” (Zhang, 2016). For differential abundance analysis between CHM and CH at the final stage (D6), we used DESeq2 (R package) applying a model that accounted for treatment effects, with a significance threshold of |log_2_ fold change| > 1 and false discovery rate (FDR) < 0.05. Because many taxa showed nominal significance (p < 0.05) but failed FDR correction, we present both the full list (**Supplementary Table 1**) and explicitly label any trends based on nominal p-values as exploratory.

### 2.4 Phytotoxicity and seedling substrate assay

Phytotoxicity was assessed by the seed germination index (GI). Ten grams of mature compost (CHM or CH) were mixed with 100 mL distilled water, shaken for 30 min, and filtered. Thirty seeds of tomato (*Solanum lycopersicum* L., cultivar ‘MicroTom’) were placed on filter paper in Petri dishes and irrigated with 5 mL of filtrate; distilled water served as control. Three replicates per treatment. After 48 h at 25 °C in the dark, germination rate (GR) and GI were calculated: GR (%) = (germinated seeds in treatment/total seeds) × 100; GI (%) = (GR_treatment_ × mean root length_treatment_) / (GR_control_ × mean root length_control_) × 100. A GI above 80% was considered indicative of non-phytotoxic and mature compost (Zucconi et al., 1981).

Based on the GI results, CHM compost was selected for substrate formulation. We tested four different blends to evaluate a range of CHM compost inclusion rates. Different mixtures (v/v) were prepared: conventional medium (CK) consisted of peat:vermiculite:perlite = 3:1:1; treatments C1 to C4 used decomposed CHM compost, decomposed cow manure, vermiculite, and perlite at ratios of 4:2:1:1 (C1), 3:2:1:1 (C2), 2:2:1:1 (C3), and 1:2:1:1 (C4) (**Supplementary Table S2**). Due to the simultaneous variation of all components, this design assesses overall blend performance rather than isolating the effect of CHM dose alone. Tomato seedlings were grown in 50-cell trays (three trays per treatment) under greenhouse conditions (25–30 °C, humidity ≥ 60%). After 40 days, shoot length, stem diameter, fresh and dry weights (shoots and roots), root to shoot ratio, and SPAD value (chlorophyll meter, Konica Minolta SPAD-502 Plus) were measured.

### 2.5 Statistical analysis

For α diversity (Shannon and Chao1 indices), Kruskal–Wallis tests followed by Dunn’s post-hoc test were used. β diversity was visualized by nonmetric multidimensional scaling (NMDS) based on Bray–Curtis distances through TUTOOL (https://www.cloudtutu.com/#/index), with stress values reported, and permutational multivariate analysis of variance (PERMANOVA, 999 permutations) used to test for significant differences between treatment and time effects. Physicochemical parameters and seedling growth data were analyzed by one-way ANOVA using SPSS 26.0. All data are presented as mean ± standard deviation.

## 3 Results

### 3.1 Microbial agent accelerated compost heating and reduced phytotoxicity

**Figure 1:**
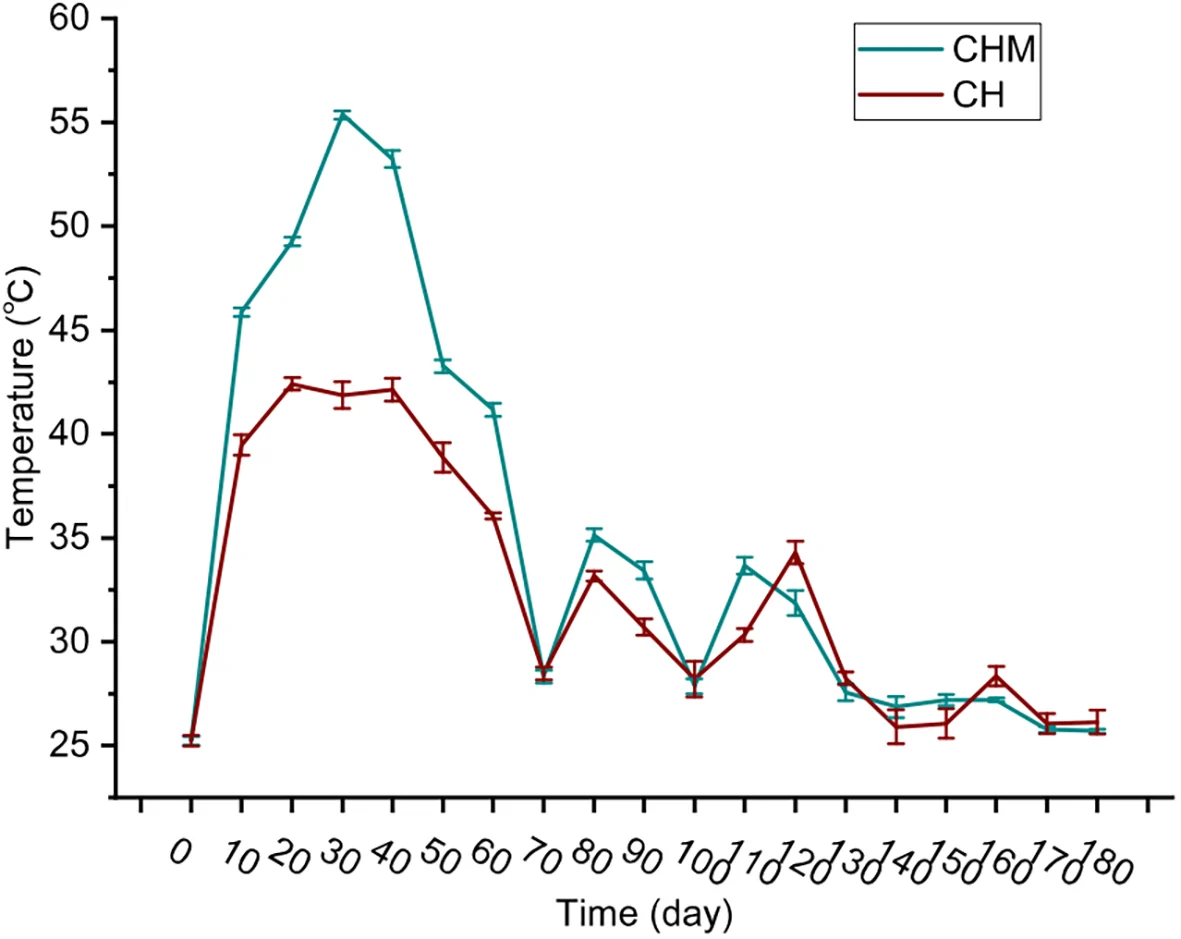
Temperature changes during composting. CHM, coffee husks with microbial agent; CH, coffee husks alone. Error bars represent standard deviations (n = 3).

**Figure 2:**
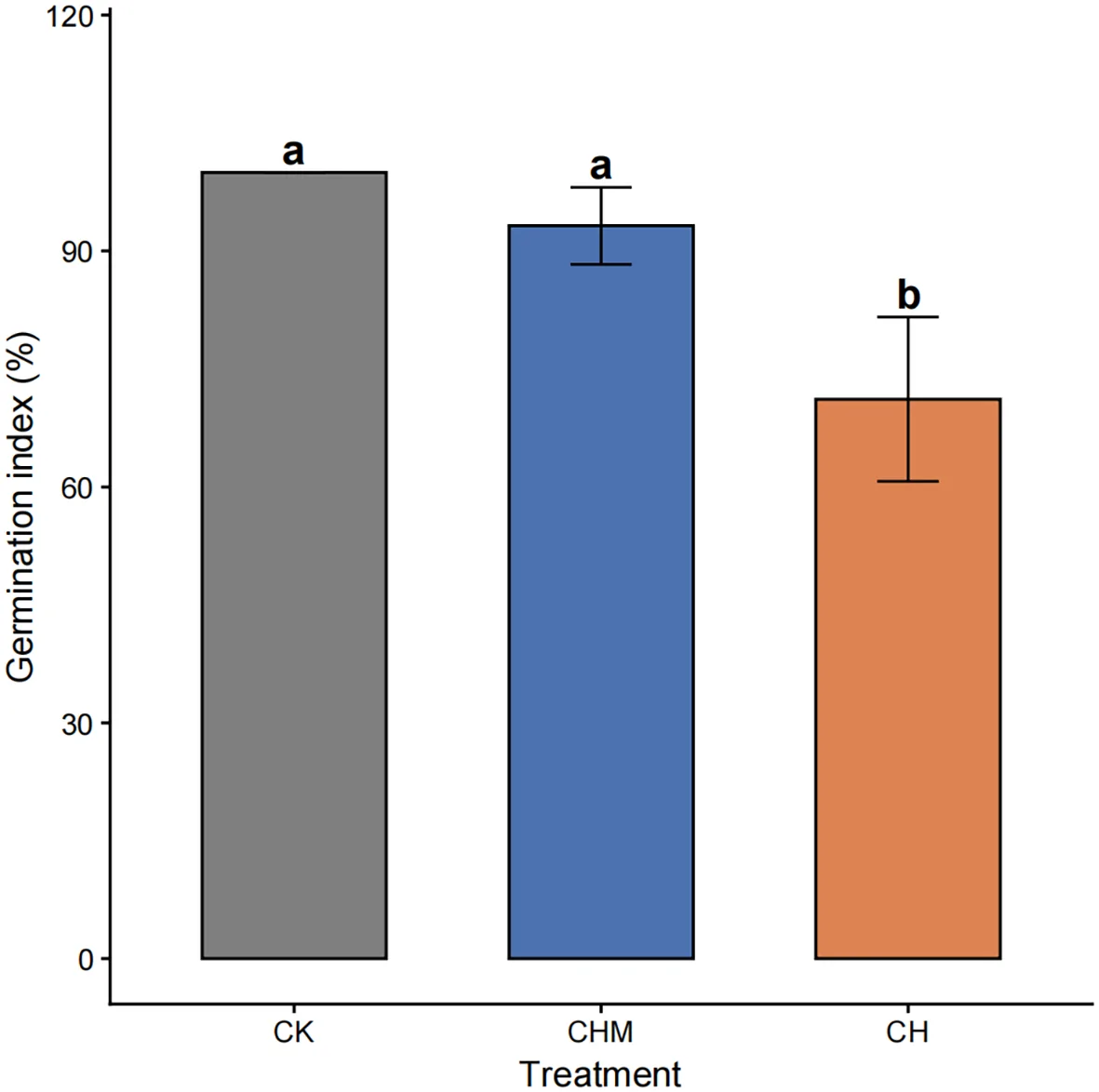
Seed germination index (GI) of mature CHM and CH composts. CK, control (distilled water). Bars represent means ± standard deviation (n = 3). Different letters indicate significant differences (ANOVA with Duncan’s test, p < 0.05).

To evaluate whether the microbial agent improved composting efficiency, we first compared temperature profiles and the seed germination index between treatments. CHM reached a peak temperature of 55.6 °C on day 30, whereas CH peaked at 43.5 °C on day 20 (**Figure 1**). The thermophilic phase (above 50 °C) lasted approximately 15 days in CHM but was not observed in CH. Both piles returned to near ambient temperature by day 170. Consistent with faster thermal processing, the GI of CHM compost (93.2%) was significantly higher than that of CH (71.2%) and did not differ from the distilled water control (100%) (**Figure 2**). A GI above 80% is generally considered non-phytotoxic and mature (Zucconi et al., 1981). Thus, CHM substantially reduced toxicity, whereas CH retained moderate phytotoxicity.

### 3.2 Microbial diversity and community succession differed between treatments

**Figure 3:**
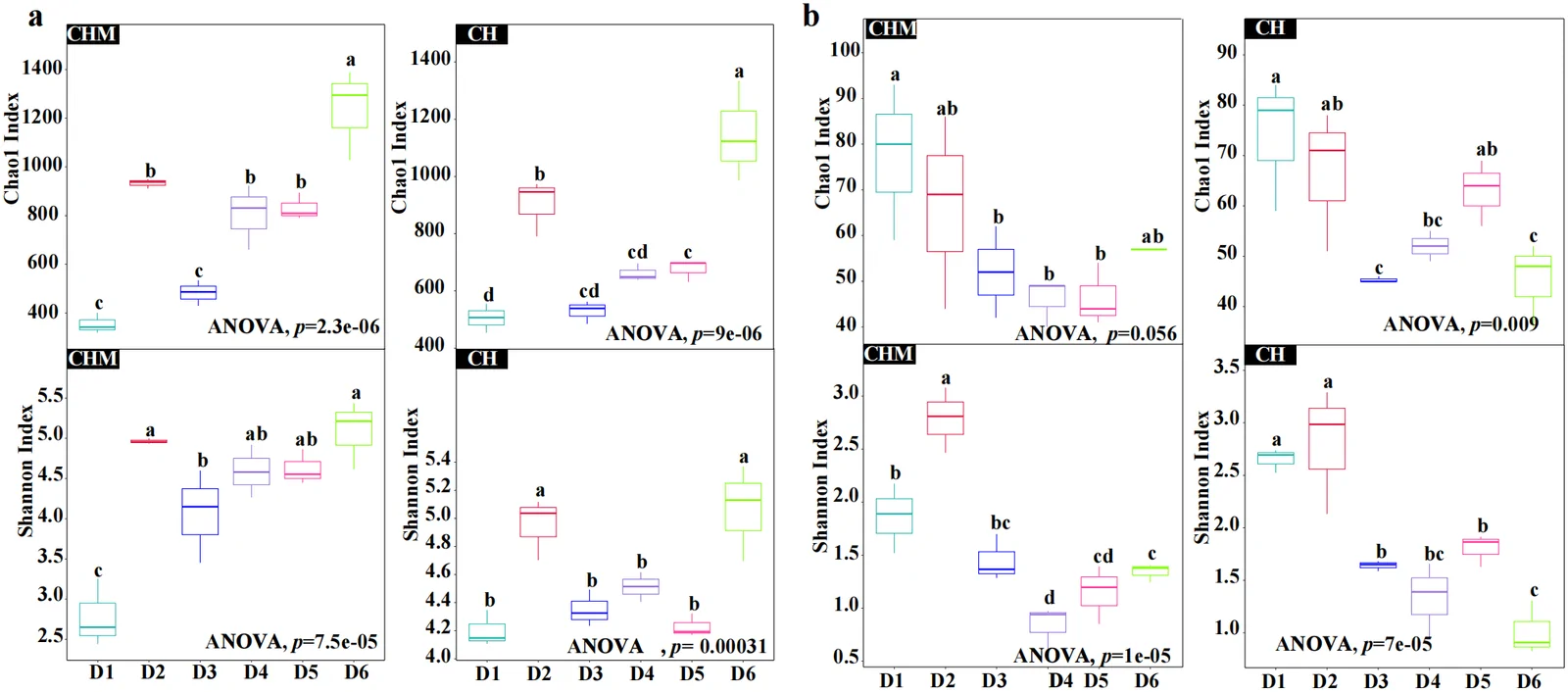
Alpha diversity of microbial communities. **(a)** Bacterial Shannon and Chao1 indices; **(b)** Fungal Shannon and Chao1 indices. CHM (coffee husks + microbial agent), CH (coffee husks alone). D1 to D6: sampling at 30, 60, 90, 120, 150, and 180 days respectively. Different letters above bars indicate significant differences.

**Figure 4:**
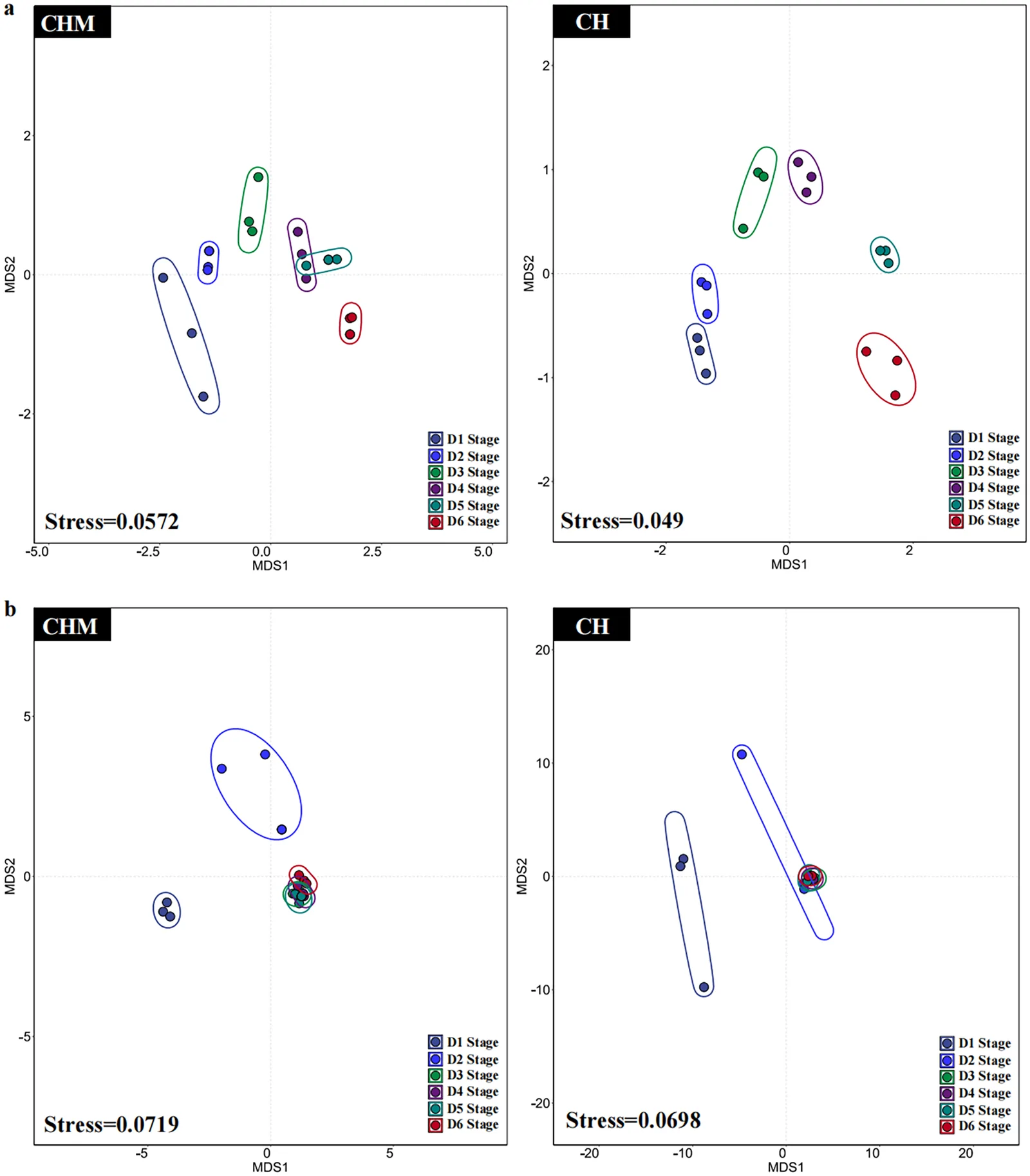
Nonmetric multidimensional scaling (NMDS) plots of microbial community composition based on Bray–Curtis distances. **(a)** Bacterial communities; **(b)** Fungal communities. Colors indicate sampling stages D1 to D6. Stress values are shown on the panels themselves.

The effect of the microbial agent on community diversity and successional trajectories was subsequently examined. Bacterial α diversity (Shannon and Chao1) increased progressively during composting in both treatments, reaching the highest values at D6 (**Figure 3a**). Fungal α diversity showed a decreasing trend over time (**Figure 3b**). At the final stage, fungal diversity was higher in CHM than in CH, indicating that the microbial agent moderated the decline. NMDS analysis revealed clear separation of bacterial communities by composting time (**Figure 4a**; stress = 0.12, PERMANOVA p < 0.001). Fungal communities at early stages (D1, D2) were distinct from later stages (D3–D6), but differentiation among D3–D5 was less pronounced (**Figure 4b**; stress = 0.15, PERMANOVA p = 0.002). Both bacterial and fungal communities at D6 were significantly different from earlier time points in each treatment. Niche width of bacteria increased with composting time (**Supplementary Figure S2a**), suggesting improved adaptation to changing environmental conditions. In contrast, fungal niche width decreased after D2 (**Supplementary Figure S2b**), implying a narrowing of habitat tolerance among fungal taxa as composting progressed.

### 3.3 CHM selectively promoted beneficial bacterial trends and was associated with lower abundance of *Fusarium*

**Figure 5:**
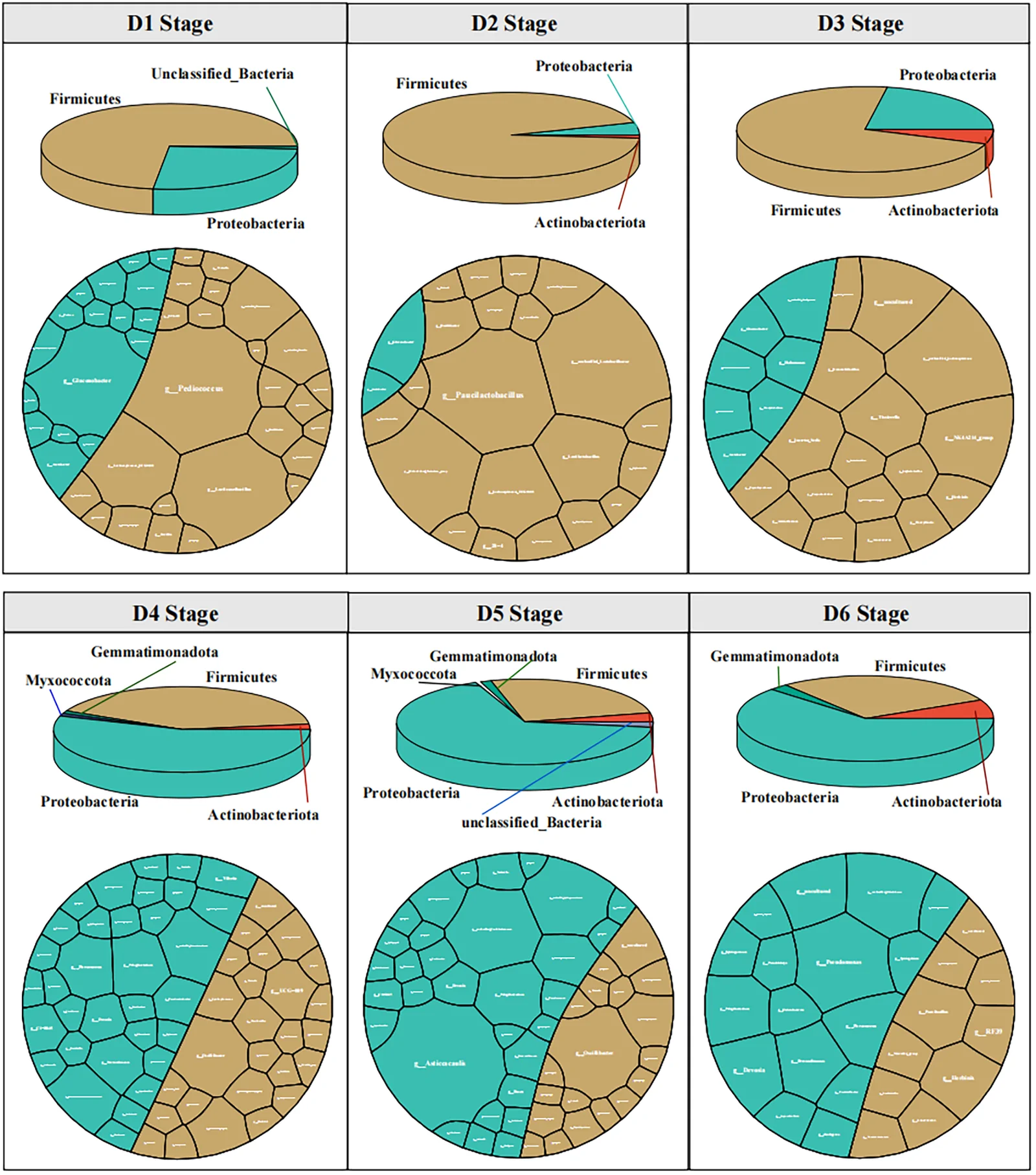
Relative abundances of dominant bacterial phyla (mean relative abundance > 1%) and their corresponding genera in the CHM and CH treatments across D1–D6. Upper panels represent phylum-level distributions; lower panels represent genus-level distributions. Different colors indicate different phyla (upper) or genera (lower), as labeled.

**Figure 6:**
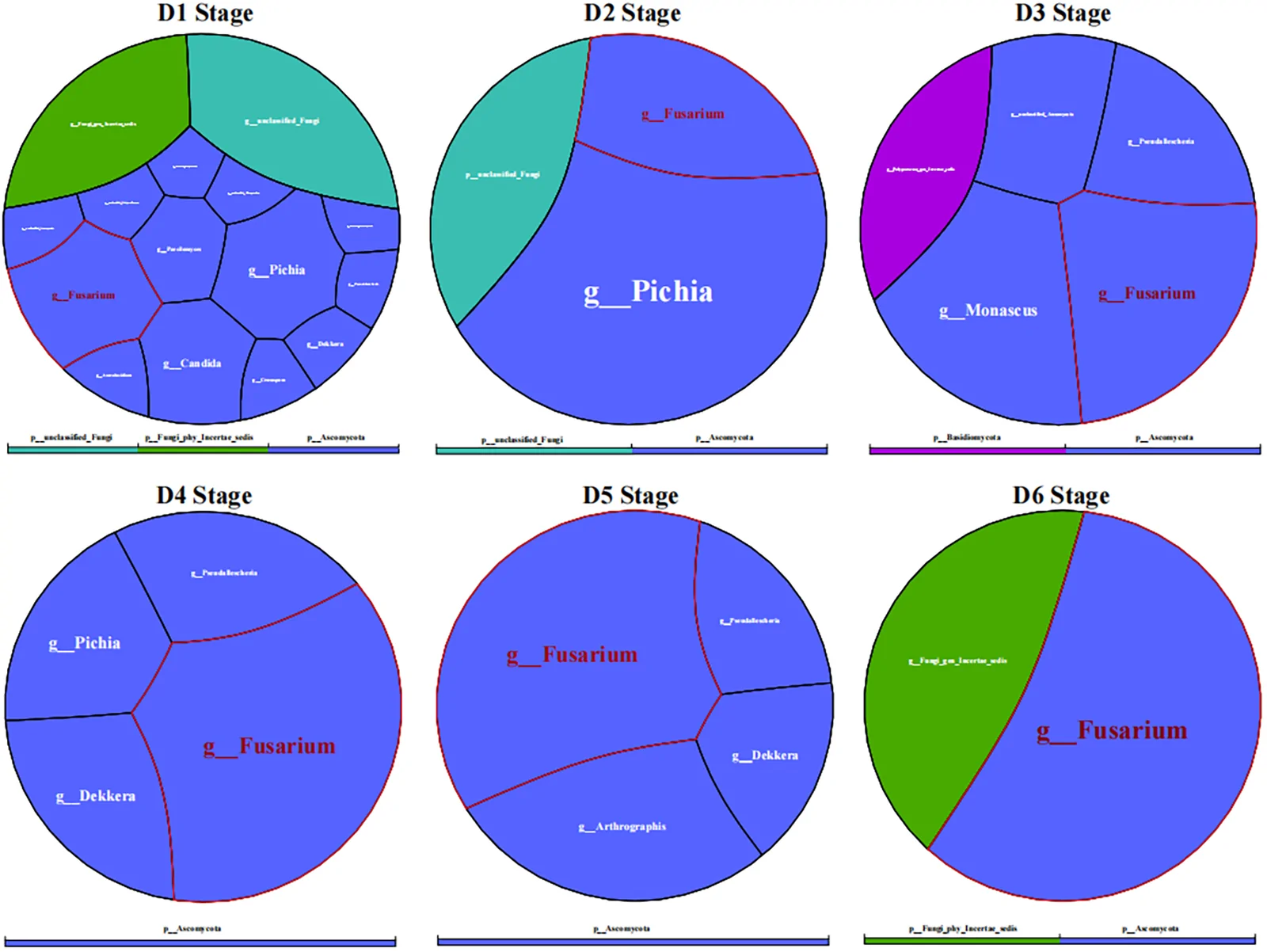
Relative abundance of dominant fungal phyla in CHM and CH treatments from D1 to D6. Only phyla with average relative abundance greater than 1% are shown. Different colors represent different phyla.

To identify which taxa responded to the microbial agent, we performed differential abundance analysis at the final stage (D6) using DESeq2. At the phylum level, Firmicutes and Proteobacteria dominated in both treatments, but Proteobacteria increased over time and became predominant in CHM at D6 (61.9%), while Firmicutes correspondingly decreased (**Figure 5**). Although strict FDR correction (FDR < 0.05) identified only a limited number of statistically significant changes (**Supplementary Table 1**), consistent directional trends emerged across replicates. Several bacterial genera displayed a trend toward higher relative abundance in CHM compared to CH as exploratory findings (nominal p < 0.05, |log2 fold change| > 3), including *Alcaligenes*, *Anaerovorax*, *Desulfuribacillus*, *Devosia*, *Paenibacillus*, *Pseudomonas*, *Sphingomonas*, and *Brevundimonas*. Many of these are known for lignocellulose degradation, xenobiotic detoxification, or plant growth promotion. In the fungal community, the relative abundance of genus *Fusarium* increased from stage D1 to D6 and became a dominant genus at the final stage (**Figure 6**). Among them, ASV_470 reached FDR significance. (nominal p < 0.05, |log2 fold change = −20.75, FDR < 0.05). (**Supplementary Table 1**; **Figure 6**). The decreasing trend of this genus suggests a potential suppressive effect of the microbial agent on this pathogen.

### 3.4 CHM based substrates supported tomato seedling growth at lower blending ratios

**Figure 7:**
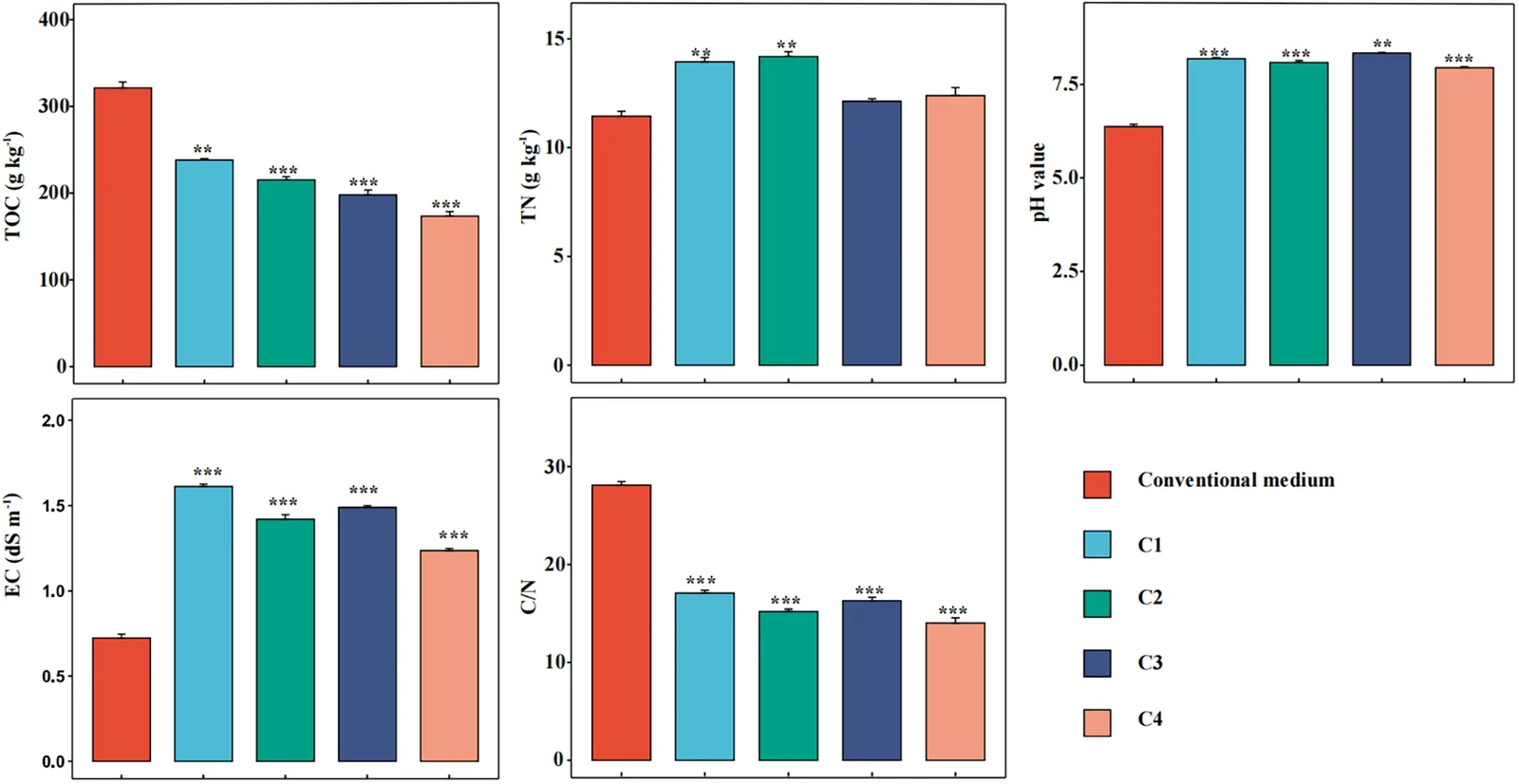
Physicochemical properties of growth media amended with different proportions of CHM compost (C1 to C4) compared to conventional peat based medium (CK). TOC, total organic carbon; TN, total nitrogen; C/N, carbon to nitrogen ratio; EC, electrical conductivity. Asterisks indicate significant differences from CK (*p < 0.05, **p < 0.01, ***p < 0.001; ANOVA with Dunnett’s test).

**Figure 8:**
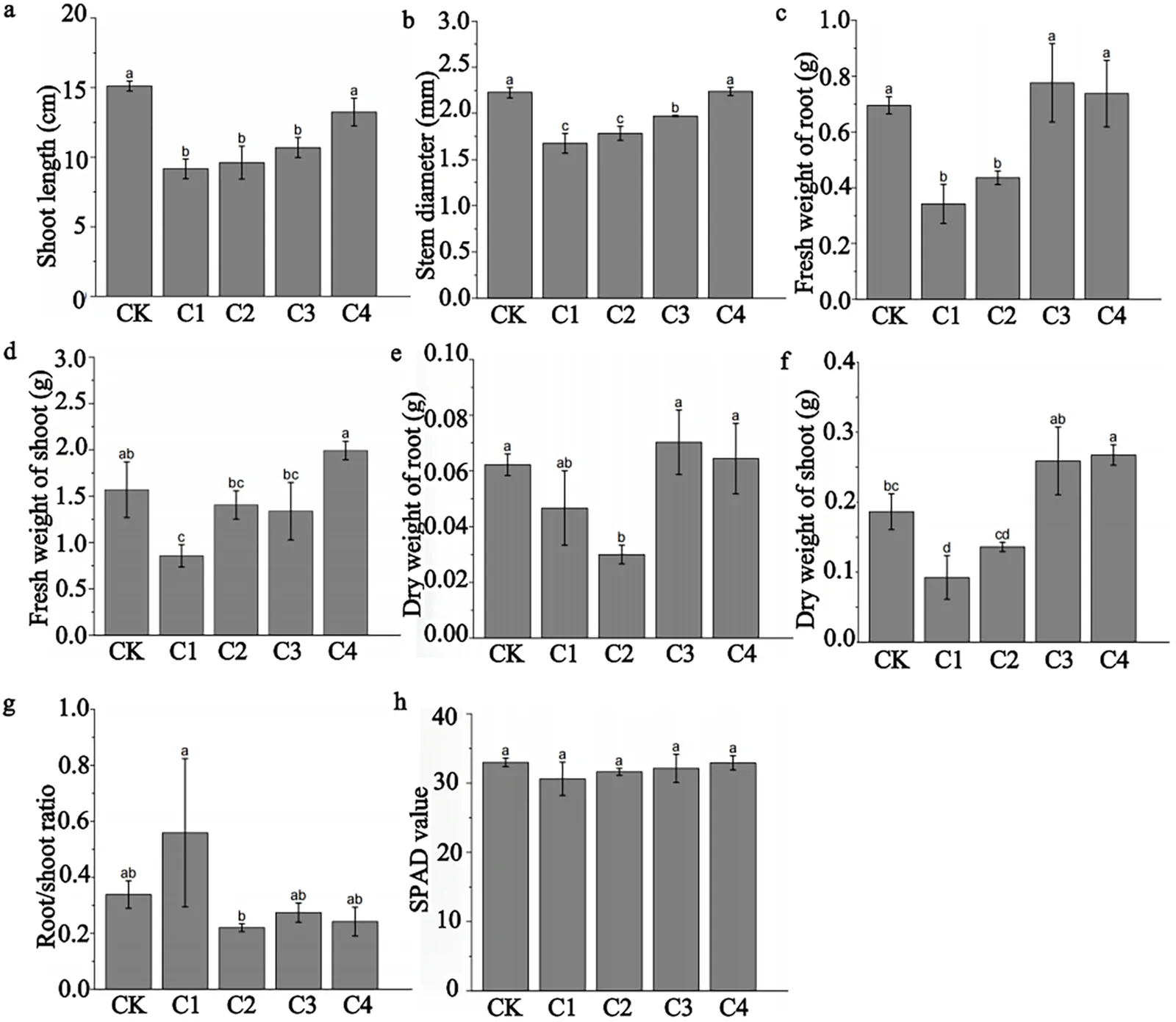
Growth parameters of tomato seedlings after 40 days on different substrates. **(a)** Shoot length; **(b)** Stem diameter; **(c)** Root fresh weight; **(d)** Shoot fresh weight; **(e)** Root dry weight; **(f)** Shoot dry weight; **(g)** Root to shoot ratio; **(h)** SPAD value. CK: conventional peat based medium; C1 to C4: increasing dilutions of CHM compost (see **Supplementary Table 2**). Error bars represent standard deviations for three replicate trays (experimental units). Same letters indicate no significant difference (ANOVA with Duncan’s test, p > 0.05). C3 (1:3 CHM blend) and C4 (1:5 CHM blend) performed similarly to CK for most parameters, whereas higher CHM proportions (C1, C2) suppressed growth, especially root development.

We then evaluated whether mature CHM compost could serve as a peat substitute. The addition of CHM compost increased pH and EC of the growth media compared to the conventional peat based medium, but all mixtures (C1 to C4) had pH between 6.2 and 7.5 and EC below 2.5 ds m^−1^, within acceptable ranges for seedling production (Bustamante et al., 2008; Gao et al., 2010). TOC and C/N decreased with higher proportions of CHM compost, whereas TN increased, particularly in C1 and C2 (**Figure 7**). After 40 days, tomato seedling growth parameters varied with the proportion of CHM compost (**Figure 8**). Shoot length, stem diameter, fresh and dry weights of roots and shoots generally increased as the CHM ratio decreased. The C4 (1:5 CHM to other components) and C3 (1:3) treatments produced seedlings that were not significantly different from those grown in the conventional peat based medium (CK) for most parameters. The C1 and C2 treatments, which contained higher proportions of CHM compost (4/8 and 3/8 v/v respectively), suppressed growth, especially root development. Root to shoot ratio and SPAD values showed no significant differences among treatments. Therefore, the CHM compost blends with lower inclusion rates (C3 and C4) showed promise as a partial peat replacement for tomato seedling substrate, when mixed with vermiculite, perlite, and cow manure.

## 4 Discussion

### 4.1 Microbial agent enhanced thermophilic phase and contributed to improved final maturity

The higher peak temperature and prolonged thermophilic phase in CHM confirm that the microbial inoculant intensified the initial decomposition of easily degradable organic matter (Yin et al., 2024). This rapid heat generation is beneficial for eliminating weed seeds and inactivating pathogens. The thermophilic phase lasting about 15 days in CHM meets sanitation requirements for compost used in horticultural applications (Shemekite et al., 2014). In contrast, the CH treatment remained mesophilic throughout, which likely contributed to slower decomposition and higher residual phytotoxicity. We hypothesize that the introduced consortium, particularly actinomycetes, which are known to secrete cellulases, hemicellulases, and lignin-modifying enzymes (Saini et al., 2015), may have contributed to degrade the recalcitrant coffee husk matrix may release heat and promote rapid temperature rise. Recent studies have shown that thermophilic bacteria such as *Geobacillus thermodenitrificans*, *Aeribacillus pallidus*, and *Ureibacillus terrenus*, isolated directly from the thermophilic phase of coffee composting, grow optimally at 60 °C and play key roles in accelerating organic matter breakdown (Papale et al., 2021). The absence of such thermophilic specialists in CH likely explains its failure to reach thermophilic temperatures. The GI results directly support this interpretation: only CHM reached the 80% maturity threshold (Zucconi et al., 1981). We propose that the detoxification effect can be attributed to enhanced degradation of phenolic acids and volatile fatty acids by the introduced consortium, particularly by *Pseudomonas* and *Sphingomonas* species, which are known to degrade aromatic compounds (Stolz, 2009; Song et al., 2022). The higher abundance trends of these genera in CHM at D6 provides indirect evidence for this hypothesized mechanism. Moreover, recent large-scale composting studies have demonstrated that microbial inoculation with strains such as *Pseudomonas* and *Planococcus* shows positive correlations with the degradation rates of cellulose, hemicellulose, and lignin, thereby accelerating the overall composting process (Wang et al., 2024).

### 4.2 Shifts in fungal communities suggest potential for pathogen suppression

A key finding of this study is the association of CHM with a marked reduction in the relative abundance of the genus *Fusarium*. *Fusarium* species are common fungal pathogens that cause wilt diseases and can persist in compost if temperatures are insufficient (Ekwomadu and Mwanza, 2023). In the CH treatment, the peak temperature of 43.5 °C was not sufficient to inactivate *Fusarium*, allowing its persistence. In CHM, the peak of 55.6 °C likely killed many propagules, but the observation cannot be explained by heat alone, as *Fusarium* chlamydospores can survive brief exposures to 55 °C (Noble and Roberts, 2004). We propose a dual-mechanism hypothesis: thermal inactivation coupled with antagonism by inoculated and indigenous bacteria. *Pseudomonas* and *Paenibacillus* secrete an array of antifungal metabolites that suppress *Fusarium* hyphal development and spore germination (Dobrzyński and Naziębło, 2024; Haas and Défago, 2005). Their consistent trend toward higher relative abundance in CHM supports this view. Additionally, the inoculated actinomycetes are well-known producers of antifungal metabolites. This hypothetical dual mechanism, thermal inactivation supplemented by biological antagonism, has been recognized as a powerful strategy for producing disease suppressive composts (Zhao et al., 2023). However, as our ASV-level differential abundance analysis showed a lack of statistical significance after correction, future studies with targeted quantification (e.g., qPCR) or metagenomics are needed to confirm active suppression. The introduction of antagonistic microorganisms such as *Bacillus mojavensis* and *Pseudomonas aeruginosa* during secondary fermentation has been shown to significantly increase antifungal activity and enhance disease control efficacy in composts (Zhao et al., 2023). Furthermore, composts provide a favorable environment for microorganisms with antagonistic potential; bacterial isolates from compost have demonstrated antifungal activity ranging from 23.3% to 83.3% against important fungal pathogens (Salman et al., 2024). More recently, the introduction of autochthonous bacterial-fungal compositions into compost has been shown to improve antagonistic activity by up to 91.7% and increase fertilizing properties by up to 35% (Mironov et al., 2023). These findings underscore the potential of microbial inoculation not only to accelerate composting but also to promote a compost microbiome with antagonistic properties, a trait that is particularly valuable in organic production systems where synthetic fungicides are restricted.

The trends of increase observed for *Alcaligenes*, *Anaerovorax*, *Devosia*, and *Desulfuribacillus* point to specialized metabolic functions. *Anaerovorax* ferments putrescine to acetate (Matthies et al., 2000), *Desulfuribacillus* participates in sulfur cycling (Sun et al., 2022), and *Devosia* is involved in xenobiotic detoxification (Talwar et al., 2020). Their increased trends suggest that the microbial agent promoted niche diversification, leading to more efficient turnover of nitrogenous and sulfur-containing compounds. Because these genera did not all pass strict FDR correction, they should be viewed as promising candidates for future targeted studies rather than definitive markers. Nevertheless, the consistent directional changes across replicates and the biological plausibility of these shifts support the conclusion that the inoculant reshaped the community in a functionally relevant manner. Notably, the agent did not dramatically alter overall α diversity but rather modulated specific functional groups, showing trends toward suppressing a potential pathogen while promoting the trend of beneficial degraders, an ecological strategy akin to “targeted microbial engineering” rather than wholesale community replacement.

### 4.3 CHM compost as a partial peat substitute: performance depends on blending ratio

Peat extraction is environmentally damaging, and renewable alternatives are urgently needed (Picca et al., 2023). Our results show that blends with lower proportions of CHM compost (C3 and C4) can serve as partial peat replacements without compromising tomato seedling quality. This performance is comparable to other agricultural waste composts (Yao et al., 2024; Ebrahimi et al., 2024).Recent studies on spent coffee grounds as peat substitutes have similarly shown that lower inclusion rates support seedling growth, with results being strongly dilution-dependent (Picca et al., 2023; Chrysargyris et al., 2021). Therefore, our data suggest that moderate blending, not pure compost, is optimal. Higher proportions (C1 and C2) suppressed growth, especially root development, likely due to excessive soluble salts or residual phytotoxic substances, despite the overall GI being acceptable. This nonlinear response underscores the importance of blending rather than using pure compost as a substrate (Capuci et al., 2024; Cangussu et al., 2021). Because our experimental design varied multiple components simultaneously, we cannot attribute performance solely to the CHM dose; rather, it identifies suitable blend formulations for future optimization. The physicochemical properties of C3 and C4 (pH 6.5–7.0, EC 1.0–1.5 dS m^−1^, adequate TN) fell within the ideal range for tomato seedling growth. Additionally, the lower relative abundance of *Fusarium* in CHM compost may confer a benefit in reducing the risk of damping-off diseases, a hypothesis that warrants direct testing in future bioassays (Cotxarrera et al., 2002; De Corato, 2020).

Several limitations should be acknowledged. Differential abundance analysis suffered from low statistical power after FDR correction, likely due to inter-replicate variation; future studies should increase replication or use metagenomics for higher resolution. We did not directly measure lignocellulolytic enzyme activities or specific phytotoxins, which would strengthen mechanistic links between microbial shifts and compost quality. The seedling experiment lasted only 40 days under greenhouse conditions; longer-term field trials are needed to validate substrate performance for full crop cycles. Finally, cost-effectiveness of the microbial agent at industrial scale requires evaluation, balancing added expense against faster turnover and improved product value. Despite these limitations, this study clearly demonstrates that microbial inoculation promotes a more robust thermophilic phase, reduces phytotoxicity, and is associated with a lower abundance of *Fusarium* in coffee husk compost, yielding a product that can partially replace peat in tomato seedling substrates. Our findings offer a practical strategy for recycling coffee processing byproducts into high-value horticultural substrates, contributing to circular agriculture. Future work should validate these results under commercial nursery conditions and optimize the microbial consortium for even greater efficiency, with particular attention to the mechanisms of pathogen suppression and enzyme-mediated lignocellulose degradation. Given the growing global interest in circular economy approaches to coffee waste valorization (Bühler et al., 2025; de Oliveira Fernandes et al., 2025), integrating microbial inoculation into coffee husk composting systems represents a promising pathway for transforming an environmental burden into a valuable resource for sustainable horticulture.

## Data Availability

The datasets presented in this study can be found in online repositories. The names of the repository/repositories and accession number(s) can be found in the article/**Supplementary Material**.
