# Supplementary material for: Microbial inoculation enhanced the thermophilic phase and improved final maturity-related properties of coffee husk composting for tomato seedling substrate production
Source: Front Plant Sci. 2026 Sep 9;17:1906101. doi: 10.3389/fpls.2026.1906101 (PMC13597231; doi:10.3389/fpls.2026.1906101)
Supplement: Supplementary file 1 [file Image1.pdf]

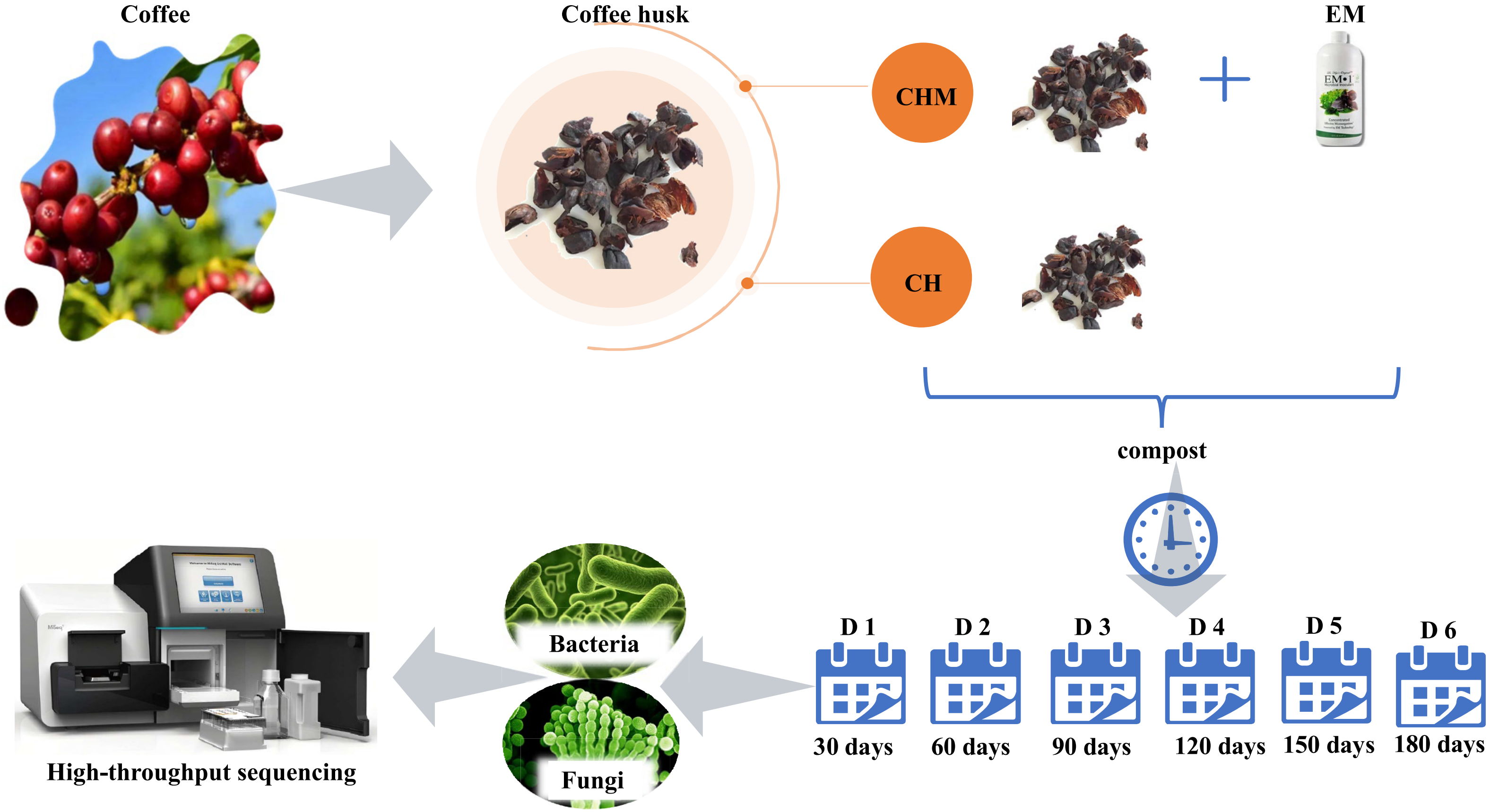

Figure S1 Flow chart of the experimental design and treatment schedule. EM represents the microbial agent, which includes photosynthetic bacteria, lactic acid bacteria, yeast, fermentation filamentous bacteria, and Actinomyces. CHM represents coffee husks + microbial agent. CH represents coffee husks alone.

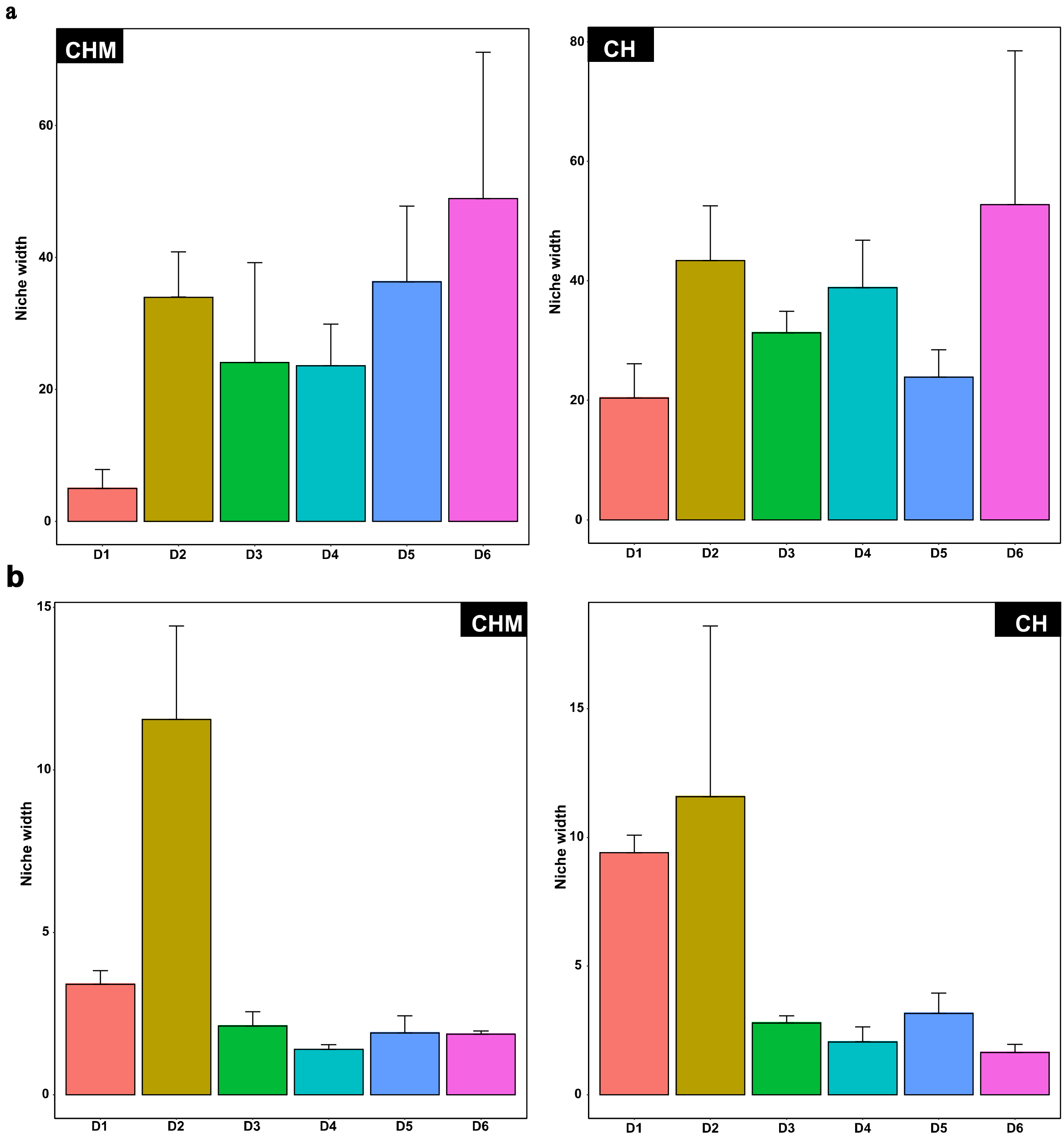

Figure S2 Niche width of microbial communities in samples at different composting times. (a) Niche width of bacterial communities. (b) Niche width of fungal communities. CHM represents coffee husks + microbial agent. CH represents coffee husks alone. D1 to D6 represent composting samples collected at 30, 60, 90, 120, 150, and 180 days, respectively.

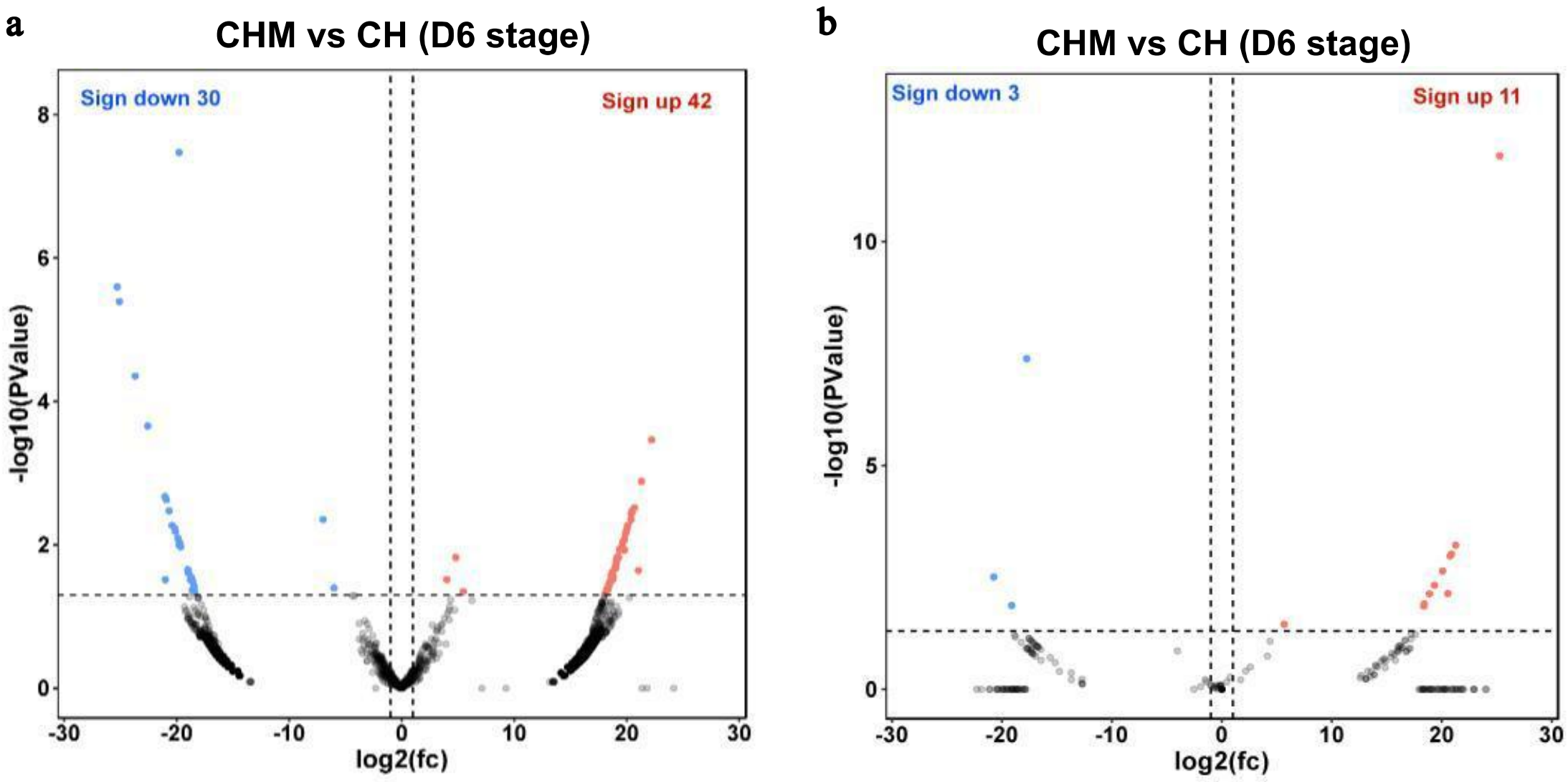

Figure S3 Comparative analysis of microbial community shifts between the CHM (coffee husks composted with a microbial agent) and CH (coffee husks alone) treatments at the final sampling stage. (a) Significant changes in bacterial community composition. (b) Changes in fungal community.
